# To Unfold the Immigrant Paradox: Maltreatment Risk and Mental Health of Racial-Ethnic Minority Children

**DOI:** 10.3389/fpubh.2021.619164

**Published:** 2021-02-17

**Authors:** Liwei Zhang, Ai Bo, Wenhua Lu

**Affiliations:** ^1^School of Social Work, Rutgers, The State University of New Jersey, New Brunswick, NJ, United States; ^2^Department of Social Work, Helen Bader School of Social Welfare, University of Wisconsin-Milwaukee, Milwaukee, WI, United States; ^3^Department of Community Health and Social Medicine, School of Medicine, City University of New York, New York, NY, United States

**Keywords:** immigrant paradox, child maltreatment, mental health, internalizing and externalizing behavior, race and ethnicity

## Abstract

Children of immigrants are often considered to be at increased risk of mental health problems due to families' immigration-related stress and perceived discrimination and prejudice from the host country. However, many studies found them to have better developmental outcomes than children with native-born parents in the U.S. This study aims to unfold this paradoxical phenomenon using data from a population-based cohort of children born in large U.S. cities. Specifically, we investigated differences in mental health outcomes between children of immigrants and those with native-born parents, stratified by children's race-ethnicity. We also explored the mediating role of child maltreatment risk in the association of parental nativity status and race-ethnicity with children's mental health. Our findings supported the immigrant paradox, with better self-reported and parent-reported internalizing and externalizing outcomes in Hispanic and Black children of immigrants than their same race-ethnicity peers and White children of native-born. Such immigrant-native variations were partially explained by parents' physically and psychologically abusive behaviors. Hispanic and Black children with immigrant parents were less likely to be physically or psychologically abused than their peers of native-born at ages 4–5, which translated into mental health advantages of children of immigrants at age 9. Our findings shed light on future research to further clarify the mechanism underlying different parenting practices between same race-ethnicity immigrants and native-born families so that culturally responsive interventions can be developed to safeguard racial-ethnic minority children's mental health.

## Introduction

In the United States (U.S.), immigration has played an essential role in shaping and reshaping the racial-ethnic diversity of children and adolescents. In 2018, ~18 million (26%) children and adolescents under the age of 18 lived with at least one foreign-born parent (i.e., children of immigrants) (1). Latinx children and adolescents constitute 24% of the U.S. population younger than 18 and will reach 34% of the country's youth population by 2060, representing the largest and fastest-growing group of ethnic minority youth (2). The Black immigrant population has also increased rapidly over the past two decades, with black immigrants and their children making up about one-fifth of the U.S. Black population (3). Children of immigrants, compared to their peers of native-born parents (hereafter, peers of native-borns), might be particularly vulnerable to mental health issues due to multiple risk factors associated with immigration, such as poverty, discrimination related to language barriers and documentation status, prejudice, and social isolation (4). However, many researchers find a noteworthy paradoxical phenomenon (i.e., the immigrant paradox) over the past 30 years: Children of immigrants generally fared better or similar in terms of mental health and behavioral outcomes than their peers of native-born, even after considering immigrant families' often disadvantaged backgrounds (5, 6). In this study, we seek to unfold this paradoxical phenomenon by investigating mental health outcomes of Black and Hispanic children of immigrants compared to their peers of native-borns. We further examine the mediating role of child maltreatment risk in the association of parental nativity status and race-ethnicity with children's mental health.

Among the large body of studies documenting the immigrant paradox, there has been an inconsistent operationalization of the paradox. Previous research often compared children of immigrants with their White counterparts with native-born parents to document the paradox (7). Such approach may miss important information on how children of immigrants fared compared with their more acculturated, same race-ethnicity peers of native-borns (7). Researchers suggested that comparisons within the same race-ethnicity could better disentangle the paradoxical phenomenon from an acculturation perspective (7, 8). For example, whereas some children have parents who just crossed the U.S. border, others may have immigration histories that extend back to four generations or higher (9). Despite having fewer resources, children of more recent immigrants often have more favorable mental health outcomes than their peers of more established immigrants (8). Such paradoxical success among children of recent immigrants implies the protective effect of certain factors related to the immigration process. It is critical, therefore, to compare the mental health of racial-ethnical minority children of immigrants with both their White peers of native-born and same race-ethnicity peers of native-born.

Studies of racial-ethnic minority often suggest that Black and Hispanic children, regardless of immigration background, experience more behavioral problems and mental health challenges than their White counterparts (10, 11). A study with a sample of children from elementary schools in Florida found more depressive symptoms among African American children compared to Euro-American peers (10). Another study examined a large sample of adolescents in grades 6–8 found that Black and Hispanic adolescents exhibited higher prevalence of internalizing and externalizing symptoms than non-Hispanic Whites (11). Several risk factors among Hispanic and Black children such as the lack of mental health care, disadvantaged socioeconomic background, and adverse childhood experience have explained the mental health disparities between White children and racial-ethnic minority children (12).

However, when distinguishing Hispanic and Black children by parental nativity status, the evidence is less straightforward. Among Hispanic children of immigrants, some research has identified that the protective effect of nativity on behavioral outcomes tend to decrease as individuals spend longer time in the United States (6, 7). A study using the National Survey on Drug Use and Health with adolescents aged 12–17 found that Hispanic immigrant adolescents were significantly less likely to be involved in externalizing behaviors than their native-born counterparts (6). Yet, in another study with a sample of Hispanic youth aged 9–17 in Chicago, children of immigrants were found to have higher internalizing behavior scores than their peers of native-borns (13). Also, most of the studies supporting immigrant paradox have focused on externalizing behaviors such as substance use and aggression (6, 14), leaving internalizing behaviors (e.g., anxiety and depression) under investigated with inconclusive findings (13, 15).

Black children and families have often been treated as a homogeneous group. Only a handful of studies examined the potential differences between immigrant and native-born Black families. A study with a nationally representative sample of children found that Black children of immigrants of age 4 compared favorably to their Black peers of native-born in terms of social behaviors in the classroom such as cooperating with other children and aggression (16). However, another study with children of age 5 in large U.S. cities found little evidence of such differences in internalizing and externalizing behaviors (17). Some research indicated that, compared to native-born Black families who have been exposed to decades of racial discrimination, recent immigrant families generally had higher socioeconomic status than their Black peers of native-born, which might have contributed to a relative advantage for their children (18–20). Other research, however, indicated that despite those advantages, Black immigrants often find it difficult to avoid disadvantaged neighborhoods and racial discrimination (21). Given the limited evidence, whether or not Black children of immigrant have a paradoxical pattern in their mental health outcomes remains unanswered.

While documenting the immigrant paradox by race-ethnicity is informative, research is also needed to identify the mechanism underlying the relations between parental nativity status and mental health outcomes among Hispanic and Black children. Parenting plays an important role in determining children's behavioral outcome and mental health functioning (22). Research indicated that the use of abusive and neglectful parenting behavior might undermine children's sense of security and give rise to emotional distress, which could lead to mental disorders in adolescence and over the life course (22–24). Young children (aged 1–6) are particularly vulnerable to maltreatment, given their dependence on caregivers during this critical developmental period (22). Both native- and foreign-born Hispanic and Black parents experience chronic stressors related to racial discrimination and structural oppression. Native-born racial-ethnic minority families, especially Black, have suffered from decades of structural violence and racial discrimination. Additionally, recent Hispanic and Black immigrant parents may experience acculturative stress due to language barriers and documentation status. These stressors may diminish parents' caregiving abilities and further disrupt their children's social and emotional functioning.

Evidence has shown that immigrant Hispanic parents are less likely to use physical punishment and emotional aggression toward their young children than native-born Hispanic parents (25–27). Some studies suggested that cultural norms on parenting may differ based on Hispanic parents' nativity status (25, 28). Compared to native-born parents, foreign-born Hispanic parents may hold stronger values of their home countries on family cohesion to protect themselves from various stressors and uncertainties related to immigration (29). The strong family values and the responsibility to care for children thus may contribute to a lower risk of maltreatment and more positive caregiving behaviors (25).

Black children, in general, are overrepresented among child abuse and neglect victims compared to Whites (30, 31). One study found a favorable pattern of parenting behaviors (e.g., breastfeeding, warm parenting) among Black immigrant parents than native-born Black parents in the U.S. (17). However, such evidence is tentative given the limited body of studies. Empirical research is needed to examine whether mental health disparities exist between children of native- and foreign-born Black parents and further clarify the role of child maltreatment risk in explaining these mental health disparities.

Taken together, children of immigrants are a heterogeneous group with vastly different social and cultural experiences. Such variety, as a determinant of parenting practices and child maltreatment risk, may complicate our understanding of the immigrant paradox. To unfold the immigrant paradox in children's mental health outcomes, it is critical to acknowledge the diversity among children of immigrants in terms of race-ethnicity and investigate the underlying mechanisms of the mental health disparities. Using a populated-based sample of U.S. families, this study investigates: (1) to what extent do mental health outcomes differ between children of immigrants and their peers of native-born across different racial and ethnic groups (i.e., non-Hispanic White, non-Hispanic Black, and Hispanic); and (2) whether child maltreatment risk, operationalized as parents' physically abusive, psychologically abusive, and neglectful behaviors, can explain racial and ethnic differences in mental health outcomes. Based on theoretical expectations and existing empirical research, we hypothesize that, across all racial-ethnic groups, children of immigrants will have more favorable mental health outcomes than their White peers of native-born and their same race-ethnicity peers of native-born. Moreover, maltreatment risk may explain the differences; that is, children of immigrants will be less likely to experience maltreatment (physical assault, psychological aggression, and neglect) than their counterparts of native-born and thus have better mental health outcomes.

## Methods

### Data

We used data from the Fragile Families and Child Wellbeing Study (FFCWS), a longitudinal study of a diverse cohort of children born between 1998 and 2000 (32). The FFCWS utilized a multistage stratified random sampling design that oversampled births to unmarried parents. Twenty large U.S. cities with populations of at least 200,000 were first sampled. Within those cities, hospitals were randomly sampled to include new births in the baseline round of data collection. Data at baseline were collected from mothers and fathers within 48 h of the child's birth, typically at the hospital. Subsequent surveys were conducted when the child was about 1, 3, 5, 9, and 15 years old. The present study used data collected at all waves except for age 15 due to the unavailability of target outcome variables.

### Sample

At baseline, the full sample included 3,711 non-marital births and 1,187 births to married parents (32). Due to attrition, the sample size was reduced to about 4,364, 4,231, 3,784, and 3,630 at age 1, 3, 5, and 9, respectively. The final sample for the analysis included 3,397 children, after restricting children to those who had valid information on outcome variables. The FFCWS oversampled births to unmarried parents, who were mostly racial-ethnic minorities, at high risk of living in poverty and experiencing child maltreatment, making it an ideal dataset for us to disentangle children's mental health variations by parental nativity status and race-ethnicity as well as the role of childhood maltreatment risk.

### Measures

#### Parental Nativity Status and Race-Ethnicity

Parents' nativity status was determined by their answers to a question regarding whether the parent was born in the United States at childbirth. Children were coded as “children of immigrants” if they had at least one foreign-born parent and were coded as “children of native-born” if both parents were born in the United States. Race-ethnicity was categorized as non-Hispanic White, non-Hispanic Black, and Hispanic based on mothers' race-ethnicity due to a large proportion of unmarried mothers in the sample. Children who did not belong to those categories (including biracial and multiracial groups) were grouped as “other” by the FFCWS. We did not include the “other” group in our analyses due to the limited racial-ethnic information. Finally, six mutually exclusive groups (i.e., non-Hispanic White of native-born, non-Hispanic Black of native-born, Hispanic of native-born, non-Hispanic White of immigrants, non-Hispanic Black of immigrants, and Hispanic of immigrants) were identified to capture the intersection of parental nativity status and race-ethnicity.

#### Internalizing and Externalizing Behavior

We operationalized mental health outcomes with internalizing and externalizing behaviors, two of the most critical mental health domains for children (5, 6). Both parent- and youth-reports at age 9 were used for children's mental health. Parent-reported scales were adapted from the Child Behavior Checklist (CBCL)/6–18 (33). Following the FFCWS's recommendations (34), we identified 32 questions from the anxiety/depression, the somatic complaints, and the withdrawal/depression subscales to examine parent-reported internalizing behaviors. Example items include “Child feels worthless or inferior” and “Child is unhappy, sad, or depressed.” The parent-reported externalizing behavior scale included 35 items from aggression and rule-breaking subscales. Example items include “Child physically attacks people” and “Child uses alcohol or drugs for nonmedical purposes.” Youth self-reported internalizing and externalizing behaviors were assessed using items from the internalizing and externalizing subscales of the Self-Description Questionnaire (35). The internalizing scale included eight items, such as “I often feel lonely,” and the externalizing scale included six items such as “I get in trouble for fighting with other kids.” The specific items included in the parent- and self-reported behavior scales were slightly different due to the differences in reporting sources. Both the parent- and self-reported scales to measure internalizing and externalizing behaviors have been widely used in previous studies with high cross-cultural validity (36–38).

For both parent- and youth-reported internalizing and externalizing behavior questions, a three-point Likert-scale was used to rate whether primary caregivers or the child felt “not true,” “sometimes or somewhat true,” or “very true” with each of the items. The responses were summed so that higher scores represent more unfavorable behaviors. In the study sample, Cronbach's alpha was 0.79 and 0.89 for parent-reported internalizing and externalizing scales, and 0.78 and 0.76 for youth-reported internalizing and externalizing scales, respectively, suggesting good reliability (34). We further computed a *Z*-score with a mean of 0 and a standard deviation of 1 to represent each child's internalizing and externalizing score relative to peers (e.g., a positive score indicates the child reported more problem behaviors than average). The correlations between parent- and child-reported mental health outcomes were 0.12 for internalizing behaviors and 0.26 for externalizing behaviors, suggesting distinct information reported from the difference sources.

In addition to the internalizing and externalizing scores collected at age 9, we used parent-reported scores collected at age 5 in our analyses (as detailed in the Analytical Strategy section) as control variables. Those scales also had good reliability for internalizing and externalizing behaviors (Cronbach's alpha = 0.88, 0.91, respectively). The FFCWS did not collect data on child-reported internalizing and externalizing behaviors at age 5.

#### Child Maltreatment

We examined childhood maltreatment risk based on primary caregivers' report on their physically abusive, psychologically abusive, and neglectful parenting behaviors toward the focal child based on items from the Parent-Child Conflict Tactics Scale (39). The physical assault subscale includes five items, such as “spanked children on the bottom with their bare hand” and “shook children.” The psychological aggression subscale contains five items, such as “shouted, yelled, or screamed at children.” The neglect subscale includes five items, such as “had to leave their children alone at home, even though they thought some adult should be with them.” Primary caregivers rated the frequency with which they had engaged in each behavior during the past year on a seven-point scale from never to more than 20 times. Following the instrument developers' recommendations (39), we recoded each individual answer to its midpoint and then averaged the answers across the items. For example, items that were endorsed “3–5 times” by primary caregivers were assigned a value of four, which is the midpoint between three and five. We next calculated *Z*-scores for physical assault, psychological aggression, and neglect, with a mean of 0 and a standard deviation of 1. We then calculated averaged scores at ages 3 and 5. The Cronbach's alpha for physical assault, psychological aggression, and neglect subscales at ages 3 and 5 were around 0.60.

#### Sociodemographic Characteristics

We considered a rich set of child and family characteristics as control variables that may influence the relationship between race-ethnicity, parental nativity status, child maltreatment, and children's mental health outcomes as identified in prior work such as by Berger (40). With the exception of child's age (available at Year 3), all covariates were from mothers' reports at baseline, including child's gender (i.e., boy or girl), whether the child had a low birth weight, mother's age at childbirth, mother's highest educational level (i.e., less than high school, high school, some college or equivalence, and bachelor's degree or above), family poverty status (i.e., below 50, 50–99, 100–199%, and above 200% of the poverty line), the number of people under age 18 in the household, and mother's romantic relationship with the child's father at childbirth (i.e., married, cohabiting, and other).

### Analytical Strategies

Missing data in the FFCWS arise from sample attrition over time and non-response. Of the analyzed sample, missing rates were <7% for all studied variables across all available time points except for parental nativity status at baseline, which had a missing rate of about 17%. We used the Full Information Maximum Likelihood (FIML) estimation in *Mplus* to account for missing values in our study (41).

**Figure 1:**
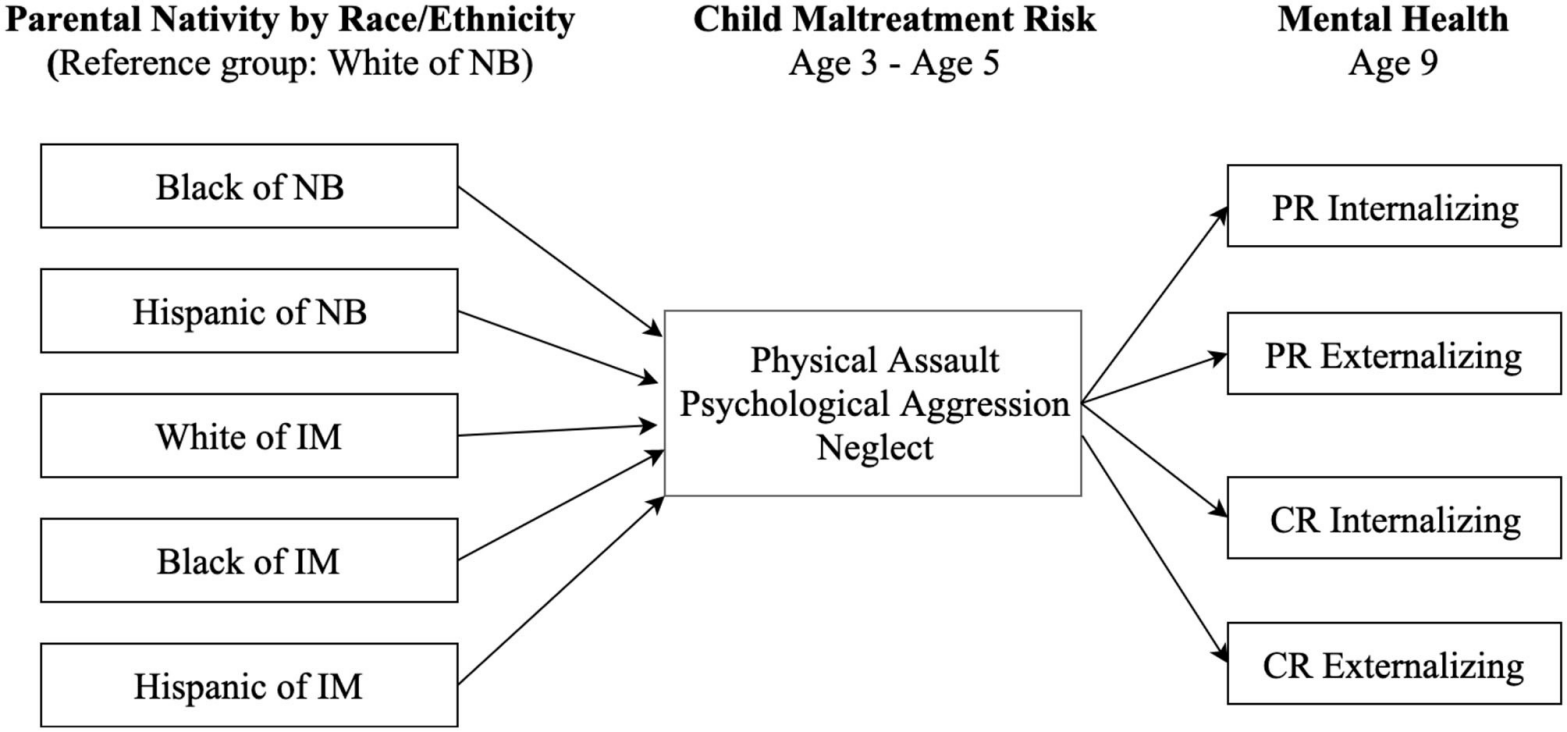
Hypothesized model depicting paths from race/ethnicity and nativity through child maltreatment risk to mental health outcomes. NB, native-born; IM, immigrant; PR, parent-reported; CR, child-reported.

We used Structural Equation Modeling (SEM) to examine mediational models in *Mplus 7.4*. As shown in Figure 1, the model entailed using path analysis to test the direct and indirect effects of race-ethnicity and parental nativity status on mental health outcomes (i.e., internalizing and externalizing) through the mediation of childhood maltreatment risk. We used three waves of data to examine the longitudinal mediation relationship, including children's race-ethnicity and parental nativity status at childbirth, maltreatment risk (mediator) at ages 3–5, and mental health outcomes at age 9. The time frames are aligned with important developmental stages from early to middle childhood and follow the evidence that parenting practice during early childhood is associated with children's later mental health functioning (22–24). Our approach also followed the methodological suggestions for longitudinal mediation (42).

All models controlled for child and family characteristics, as indicated above, to partial out the omitted variable bias to a certain degree. Because physical assault and psychological aggression were highly correlated (*r* = 0.73, *p* < 0.001), following prior research (43), we conducted three separate path analyses for physical assault, psychological aggression, and neglect, respectively, to address the conceptually distinct yet highly correlated nature of the maltreatment types.

An essential component of mediation analysis using longitudinal data is to residual the dependent variables so that we can determine whether race-ethnicity and parental nativity status were associated with changes in children's internalizing and externalizing behaviors between age five and age nine through childhood maltreatment risk (42). We thus also controlled for children's internalizing and externalizing behaviors reported by primary caregivers that were measured at earlier waves (age 5). We evaluated model fit by examining a variety of goodness-of-fit indices established by Hu and Bentler (44). A good data-model fit should have a standardized root mean square residual (SRMR) ≤ 0.08, a root mean square error of approximation (RMSEA) ≤ 0.08, and a comparative fit index (CFI) ≥0.90.

## Results

### Descriptive Statistics

Table 1 displays descriptive statistics for the sampled children by parental nativity. Of the sampled children (*N* = 3,397), about 21% had at least one parent who was born outside of the United States. Among children of native-born, about 26% were non-Hispanic White, 55% were non-Hispanic Black, and 19% were Hispanic. Of the children of immigrants, about 11% were non-Hispanic White, 18% were non-Hispanic Black, and 71% were Hispanic. We found that children of immigrants were less likely to have a low birth weight and more likely to live in a married family, while more likely to have worse family socioeconomic status than their peers of native-born. Our results indicate that children of immigrants had a lower risk of physical assault and psychological aggression, comparable neglect score, better parent-reported externalizing, and better self-reported internalizing and externalizing behavior scores than their peers of native-born.

**Table 1:** Descriptive statistics for sampled children by parental nativity status (*N* = 3,397).

	**Native-born**	**Immigrant**	**Total**
Parental nativity status (%)	79.50	20.50	100.00
**Race/ethnicity (%)**			
Non-hispanic white	26.62	10.83	22.94
Non-hispanic black	54.67	17.86	46.08
Hispanic	18.71	71.32	30.98
Gender (% boy)	52.49	51.72	52.31
Low birth weight (%)	11.44	5.40	10.03
Mother's age (years) at wave 1 (Mean, SD)	25.01 (6.06)	26.34 (6.04)	25.32 (6.08)
Child's age (months) at wave 2 (Mean, SD)	35.60 (2.41)	36.11 (2.81)	35.71 (2.51)
**Mother's romantic relationship with father at childbirth (%)**			
Married	23.79	34.71	26.34
Cohabitating	41.53	41.16	41.45
Other	34.68	24.14	32.21
No. of household members <18 (Mean, SD)	1.28 (1.31)	1.12 (1.24)	1.24 (1.29)
**Mother's highest education level (%)**			
Less than high school	30.19	51.22	35.10
High school	33.07	19.93	30.00
Some college and equivalence	25.20	19.38	23.84
Bachelor's degree and above	11.54	9.47	11.06
**Household income (% of federal poverty line)**			
200+	41.64	32.93	39.60
100–200	24.30	29.92	25.61
50–100	15.88	20.91	17.06
<50	18.19	16.24	17.73
**Maltreatment risk (*****Z*****-scores)**			
Physical assault	0.11 (1.05)	−0.37 (0.63)	0.00 (0.98)
Psychological aggression	0.13 (1.02)	−0.39 (0.77)	0.01 (0.99)
Neglect	−0.01 (0.86)	0.00 (1.34)	−0.01 (1.00)
**Mental health outcomes (*****Z*****-scores)**			
Parent-reported internalizing	−0.01 (0.46)	0.05 (0.49)	0.00 (0.47)
Parent-reported externalizing	0.00 (0.52)	−0.07 (0.48)	−0.01 (0.52)
Youth-reported internalizing	0.02 (0.62)	−0.08 (0.58)	0.00 (0.62)
Youth-reported externalizing	0.03 (0.68)	−0.18 (0.59)	−0.01 (0.67)

*Percentages are shown for categorical variables; Mean and standard deviations (in parenthesis) are shown for continuous variables*.

### Paths From Race-Ethnicity and Parental Nativity Through Maltreatment to Mental Health

**Figure 2:**
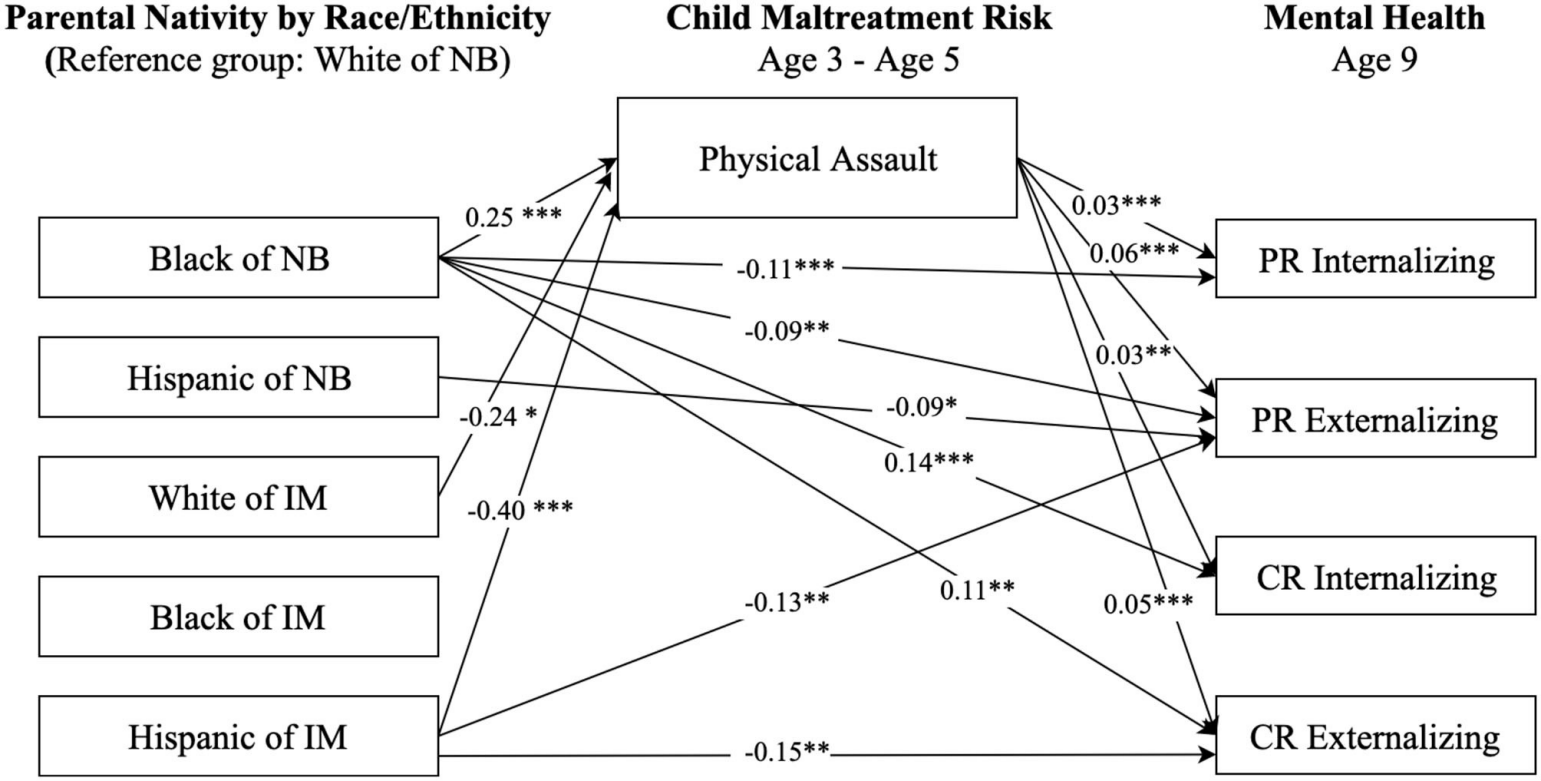
Paths from race/ethnicity and parental nativity through child physical assault risk to internalizing and externalizing behaviors. NB, native-born; IM, immigrant; PR, parent-reported; CR, child-reported. Numbers shown are statistically significant coefficients. Model estimates were derived from structural equation modeling analysis controlling for sociodemographic characteristics as described in the Methods section. RMSEA = 0.056, CFI = 0.891, and SRMR = 0.027. *p < 0.05, **p < 0.01, and ***p < 0.001.

**Figure 3:**
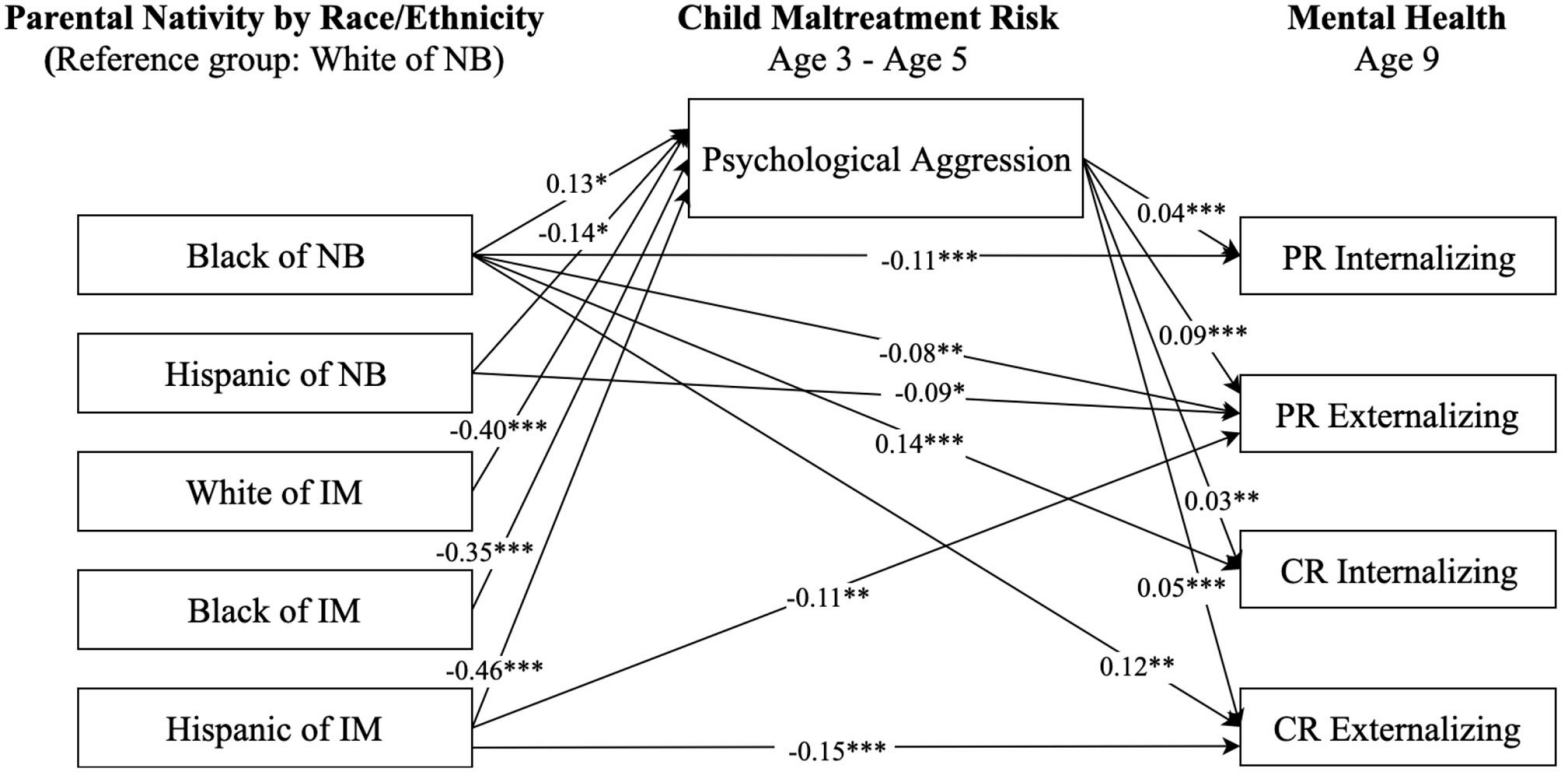
Paths from race/ethnicity and parental nativity status through child psychological aggression risk to internalizing and externalizing behaviors. NB, native-born; IM, immigrant; PR, parent-reported; CR, child-reported. Numbers shown are statistically significant coefficients. Model estimates were derived from structural equation modeling analysis controlling for sociodemographic characteristics as described in the Methods section. RMSEA = 0.062, CFI = 0.871, and SRMR = 0.029. *p < 0.05, **p < 0.01, and ***p < 0.001.

**Figure 4:**
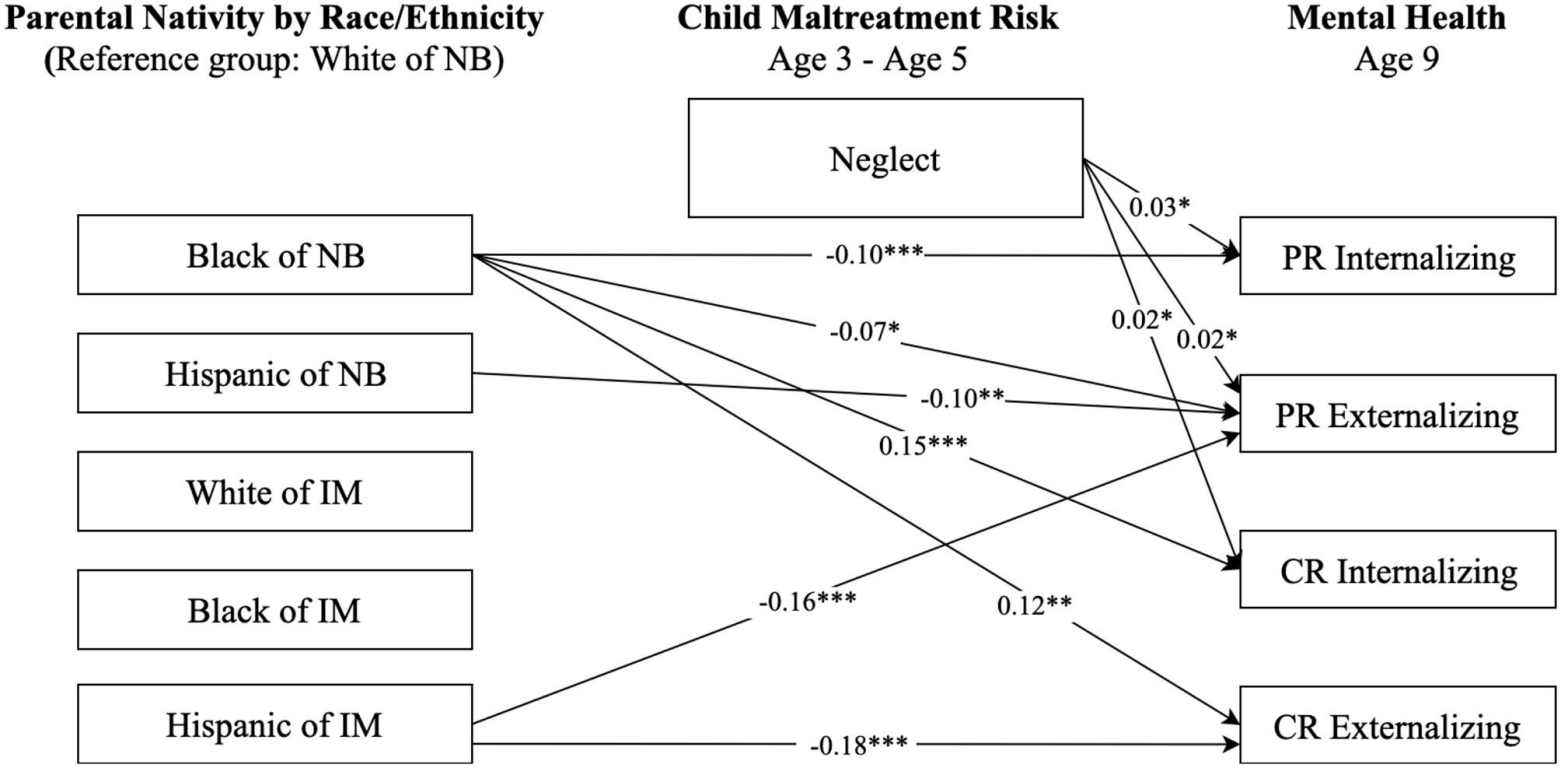
Paths from race/ethnicity and parental nativity through child neglect risk to internalizing and externalizing behaviors. NB, native-born; IM, immigrant; PR, parent-reported; CR, child-reported. Numbers shown are statistically significant coefficients. Model estimates were derived from structural equation modeling analysis controlling for sociodemographic characteristics as described in the Methods section. RMSEA = 0.049, CFI = 0.931, and SRMR = 0.025. *p < 0.05, **p < 0.01, and ***p < 0.001.

Figures 2–4 display statistically significant parameter estimates for the structural model examining paths from race-ethnicity and parental nativity status through child maltreatment risk to internalizing and externalizing behaviors. The reference group for race-ethnicity and parental nativity is White children of native-born. Model fit indices suggested acceptable model fit (e.g., RMSEA = 0.056, CFI = 0.891, and SRMR = 0.027, as shown in Figure 2).

Results in Figure 2 indicate significant direct and indirect associations between race-ethnicity and parental nativity groups and children's internalizing and externalizing behaviors, after controlling for child and family characteristic variables (e.g., mother's highest educational level, family poverty status). Regarding direct relationships, Black children of native-born had lower (better) parent-reported internalizing (*b* = −0.11, *p* < 0.001) and externalizing (*b* = −0.09, *p* < 0.05) scores compared to White children of native-born. However, Black children of native-born had higher (worse) self-reported internalizing (*b* = 0.14, *p* < 0.001) and externalizing (*b* = 0.11, *p* < 0.01) scores compared to the White children of native-born. Hispanic children of native born had better parent-reported externalizing scores (*b* = −0.09, *p* < 0.05) than White children of native-born. Hispanic children of immigrants had better parent-reported externalizing scores (*b* = −0.13, *p* < 0.01) and self-reported externalizing scores (*b* = −0.15, *p* < 0.05) than their White peers of native-born.

Regarding indirect relationships, as shown in Figure 2, physical assault significantly mediated the association between race-ethnicity and parental nativity and children's internalizing and externalizing behaviors. Specifically, Black children of native-born had a higher risk of experiencing physical assault (*b* = 0.25, *p* < 0.001) than White children of native-born. Hispanic children of immigrants had a significantly lower risk of physical assault (*b* = −0.40, *p* < 0.001) relative to White children of native-born. The risk of experiencing physical assault, in turn, was positively associated with levels of parent- and self-reported internalizing and externalizing behaviors.

Figure 3 presents the paths from race-ethnicity and parental nativity through psychological aggression risk to internalizing and externalizing behaviors. The patterns of direct associations in Figure 3 were similar to those in Figure 2. Regarding indirect associations, children from all race-ethnicity and parental nativity groups, except for Black children of native-born, had a significantly lower risk of psychological aggression, and in turn, lower parent- and self-reported internalizing and externalizing behaviors compared to White children of native-born.

Results in Figure 4 only show statistically significant direct paths from race-ethnicity and parental nativity status to internalizing and externalizing behaviors. No significant paths were found from race-ethnicity and parental nativity to neglect, though there were significant paths from neglect to mental health outcomes, suggesting no significant mediating pathways through neglect in the relationship between race-ethnicity and parental nativity and internalizing and externalizing behaviors.

### Mental Health Paradox Among Hispanic and Black Children of Immigrants

**Figure 5:**
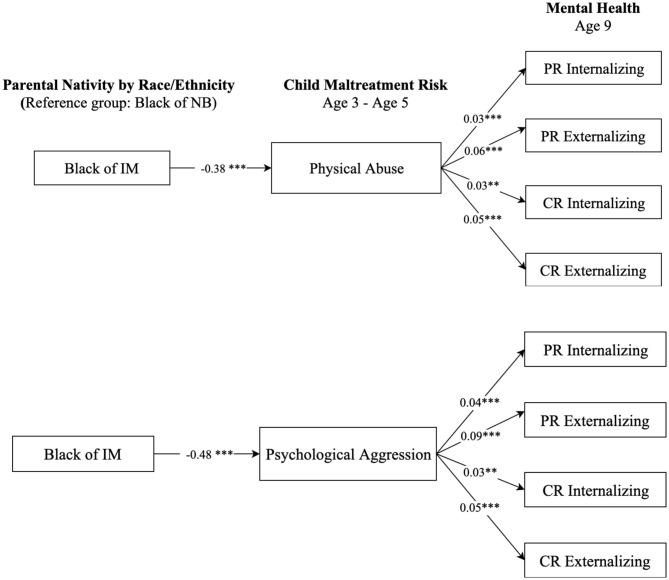
Paths from parental nativity through child maltreatment risk to internalizing and externalizing behaviors for non-Hispanic Black children. NB, native-born; IM, immigrant; PR, parent-reported; CR, child-reported. Numbers shown are statistically significant coefficients. Model estimates were derived from structural equation modeling analysis controlling for sociodemographic characteristics as described in the Methods section. RMSEA = 0.056, CFI = 0.891, and SRMR = 0.027, for the model with physical assault. RMSEA = 0.062, CFI = 0.871, and SRMR = 0.029, for the model with psychological aggression. **p < 0.01, and ***p < 0.001.

**Figure 6:**
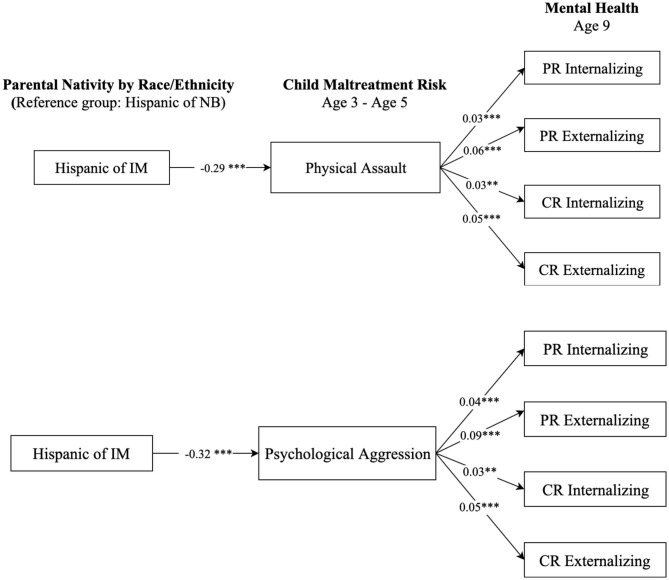
Paths from parental nativity through child maltreatment risk to internalizing and externalizing behaviors for Hispanic children. NB, native-born; IM, immigrant; PR, parent-reported; CR, child-reported. Numbers shown are statistically significant coefficients. Model estimates were derived from structural equation modeling analysis controlling for sociodemographic characteristics as described in the Methods section. RMSEA = 0.056, CFI = 0.891, and SRMR = 0.027, for the model with physical assault. RMSEA = 0.062, CFI = 0.871, and SRMR = 0.029, for the model with psychological aggression. **p < 0.01, and ***p < 0.001.

We further examined whether racial and ethnic minority children of immigrants, namely non-Hispanic Black and Hispanic children, had a lower maltreatment risk and thus better mental health outcomes relative to their peers of native-born. Figure 5 presents the results comparing children of native-born and immigrants who are non-Hispanic Black, and Figure 6 displays comparison results between Hispanic children of native-born and their peers of immigrants. The results in Figure 5 show that, compared to Black children of native-born, Black children of immigrants had a significantly lower risk of being physically and psychologically abused (*b* = −0.38, *p* < 0.001; *b* = −0.48, *p* < 0.001, respectively). Such differences contributed to more favorable mental health scores for Black children of immigrants compared to their peers of native-born. Results in Figure 6 show similar patterns regarding the differences between Hispanic children of native-born and their peers of immigrants on physical assault (*b* = −0.29, *p* < 0.001) and psychological aggression (*b* = −0.32, *p* < 0.001). Such results suggest that Black and Hispanic children of immigrants were less likely to suffer from maltreatment, which contributed to better mental health outcomes than their peers of native-born with same race-ethnicity.

## Discussion

To unfold the immigrant paradox in children's mental health outcomes, this study investigated factors that can potentially explain mental health disparities based on race-ethnicity and parental nativity status. Specifically, we examined the role of child maltreatment risk (i.e., physical assault, psychological aggression, and neglect) in mediating the associations of parental nativity status and race-ethnicity with children's internalizing and externalizing outcomes. Our findings supported an immigrant paradox in children's mental health outcomes, suggesting that children of immigrants generally had more favorable self-reported and parent-reported internalizing and externalizing scores than both White children of native-born and their same race-ethnicity peers of native-born. Such native-immigrant variations were partially explained by parents' physically and psychologically abusive behaviors. More specifically, Hispanic and Black children of immigrants were less likely to be physically or psychologically abused than their same race-ethnicity peers of native-born at ages 4–5, and thus had better internalizing and externalizing outcomes at age 9. We discuss our findings separately for Hispanic and non-Hispanic Black children below considering the unique cultural characteristics with each group.

Our findings suggest that having mothers living in and acculturating to the U.S. culture may be associated with increased risk of maltreatment and, in turn, mental health difficulties among Hispanic children of native-born compared to their peers of immigrants. Such results are consistent with existing studies suggesting that first- and second-generation Hispanic children show better mental health outcomes than their peers of U.S.-born parents from comparable socioeconomic backgrounds (5, 13). One explanation of such differences is parenting differences due to acculturation levels. Foreign-born Hispanic parents are found to adhere more to cultural values and connections to their countries of origin, which may protect parents from experiencing parenting stress (26, 45). For example, the Hispanic culture *familismo* values closeness and interconnectedness among extended family members, which includes a sense of responsibility to care for family members, particularly for young children (46). Recent Hispanic immigrant parents with strong family values may want to protect their children from potential harms of the host country by providing stronger parental supervision and support. Also, due to limited access to resources and barriers related to documentation status and language difference, foreign-born parents are likely to develop close family networks and extended social support within their immigrant communities, all of which may indirectly protect their children from maltreatment and protect children's mental health functioning (25, 29).

The immigrant paradox in children's mental health was also explored among non-Hispanic Black children in this study. Compared with Black children of native-born, Black children of immigrants had better parent- and youth-reported internalizing and externalizing outcomes. Compared to studies on Hispanic immigrants, research in the “Black immigrant paradox” is noticeably lacking. In particular, there is little evidence on the mental health advantages among Black children of immigrants. Some research suggested that although immigrant and native-born Black families may experience comparable socioeconomic disparities and racial discrimination, immigrant cultural traits such as family cohesion and positive parenting could serve as a buffer against those adverse conditions for Black immigrant parents (16, 17). Our results aligned with such evidence, suggesting that Black immigrant parents were less likely to engage in physically and psychologically abusive parenting than native-born Black parents, which prevented their children from experiencing mental health difficulties. Previous studies indicated that perceived discrimination increases as people spend more time living in the United States (47, 48). Native-born Black parents have been exposed to decades of structural racism rooted in slavery in the U.S. As native-born parents experience and perceive more discrimination acts, it is likely that they are more mentally distressed and have poorer parenting abilities compared to foreign-born parents who perceived less discrimination acts. Given the parenting and mental health differences by parental nativity status, mental health prevention and treatment programs targeting Black children need to understand the similarities (e.g., the experience of discrimination, socioeconomic disadvantage) and differences (e.g., cultural and language variations) between native-born and immigrant Black families and provide targeted supports to address their respective challenges and needs.

Additionally, we found that Black children of native-born reported themselves to have the most internalizing and externalizing difficulties among all groups, which is consistent with existing evidence suggesting the disproportionate burden of mental health disorders among Black children (12, 49). However, with respect to parent-reports, those children were reported to have better internalizing and externalizing scores than White children of native-born. The discrepancies between child-reported and parent-reported mental health outcomes of Black children provide some insights of mental health disparities. Previous studies have reported low to moderate parent-child agreement on children's emotional and behavioral problems, with correlations ranging from 0.25 to 0.44 and kappa values between 0.04 and 0.29 (50–52). In our sample, the correlations between parent- and child-reported mental health outcomes were 0.12 for internalizing behaviors and 0.26 for externalizing behaviors, suggesting low parent-child agreement. Compared with parents, children are suggested to be more likely to recognize their mental health problems and be more sensitive to their social and emotional difficulties in interacting with other children and thus report higher severity ratings (50, 53). This could be particularly true for Black children who experience socioemotional challenges due to perceived racial discrimination and prejudice during their early schooling years (54). Moreover, parents who engage in abusive parenting activities are likely to neglect their children's mental health needs (55). Given the high degrees of physical and psychological abuse risk among native-born Black parents, it is plausible that their children perceived higher levels of mental health difficulties than their parents.

Although our findings suggest the potential protective role of parental cultural values in immigrant families, it is unclear how gaining American cultural values could contribute to the immigrant paradox in children's mental health. Evidence on the role of the U.S. culture has been mixed. On the one hand, the U.S. culture can be protective because it does not endorse authoritarian beliefs and practices as much as in other cultures; such less restrictive parenting style may contribute to lower risk of physical punishment (56). On the other hand, research suggests that acculturation gaps between parents and children could contribute to conflicts and thus increase the risk of children's mental health problems (57, 58). Given the mixed findings, future research needs to examine how the acculturation processes in the immigrant community, such as identification with one's ethnic group and with the mainstream U.S. society, can drive the differences in maltreatment and mental health between children of foreign-born and native-born parents.

### Limitations

Several caveats apply to the findings presented in this study. First, we were not able to include other racial-ethnic groups such as Asian, Native American, and children with mixed-racial backgrounds in our analyses due to the unavailability of such data. National data suggested that the number of newly arrived Asian immigrants have surpassed Hispanic immigrants since 2010 (59). However, nationally representative data is limited in tracking this increasingly growing immigrant group. Also, it is documented that Native American children and adolescents have the highest self-reported depression rates among all racial-ethnic groups (60). Given the unique cultural values and parenting practices among each racial-ethnic group, further research is needed to understand the complex ways of parenting and child maltreatment risk in shaping children's mental health in each racial-ethnic group. Second, we were only able to categorize immigrant groups by race-ethnicity due to the data at hand. Within the same racial-ethnic group, immigrants are diverse in terms of language, culture, generational status, country of origin, and time of migration, which could translate into variations in parenting and children's mental health (61). Future studies should examine subgroup differences within each racial-ethnic group with more in-depth measures. Third, although we controlled for a series of variables related to child and family characteristics in our analysis, we recognize that there were uncontrolled factors (e.g., parental mental health and substance use, neighborhood deprivation, and region of residence) that may have resulted in residual confounding. Detailed measures for family socioeconomic status are also needed in future studies. Fourth, child maltreatment risks were measured based on parents' self-report, which is subject to social desirability bias (62). Fifth, our analytic samples were overrepresented by single-parent families who resided in large U.S. cities. Therefore, our findings may not be generalizable to the entire population of U.S. families or families in rural areas. Nevertheless, the sampling strategy by FFCWS allowed us to explore the mental health of children from “non-traditional families” who are at high risk of living in poverty and would have been less likely to participate under other circumstances (63). Finally, the relationship between child maltreatment and mental health outcomes can be reciprocal, where children with mental health difficulties are more likely to be maltreated (64), and the maltreatment may further worsen the children's mental health outcomes. Further research is needed to examine the reciprocal relationship.

## Conclusions

Despite the limitations, this study's results provide substantial evidence on the immigrant paradox in children's mental health among Black and Hispanic children and the underlying mechanisms through child maltreatment risks. The relatively better mental health outcomes among immigrant children do not suggest that they need less mental health prevention and treatment services. Instead, children of immigrants that need mental health services are found to be less likely to receive treatment than their peers of native-born due to a wide range of structural and sociopolitical obstacles, such as poverty, lack of insurance, language, and cultural barriers, and insufficient availability of mental health services in immigrant neighborhoods (12). The U.S. population has undergone a rapid demographic change in racial and ethnic compositions from two decades ago. In many parts of the country, the traditional “racial-ethnic minority” has become the majority. Our sampled children were born in 1999–2000 and the generation is now in their adult years and will soon (or may have already) become parents. Thus, understanding their maltreatment risk during early childhood and later mental health outcomes provides insight into the life course trajectory of a generation of young Black and Hispanic adults and future (or current) parents. Our findings could inform culturally sensitive strategies that can boost immigrant families' strengths (e.g., lower maltreatment risk, strong family values) and address their challenges (e.g., limited access to mental health services, language, cultural, and structural barriers).

## Data Availability Statement

Publicly available datasets were analyzed in this study. This data can be found here: https://fragilefamilies.princeton.edu/data-and-documentation/public-data-documentation.

## Ethics Statement

Ethical review and approval was not required for the study on human participants in accordance with the local legislation and institutional requirements. Written informed consent to participate in this study was provided by the participants' legal guardian/next of kin.

## Author Contributions

LZ contributed to the study design and performed the data analysis. All authors, including LZ, AB, and WL interpreted the results and contributed to the final manuscript.

## Conflict of Interest

The authors declare that the research was conducted in the absence of any commercial or financial relationships that could be construed as a potential conflict of interest.
